# Prediction of attention deficit hyperactivity disorder using the comprehensive attention test: a large-scale machine learning approach

**DOI:** 10.3389/fpsyt.2025.1574615

**Published:** 2025-05-27

**Authors:** Kwang Su Cha, Bongseog Kim, Jun-Young Lee, Hanik Yoo

**Affiliations:** ^1^ Corporate Research Institute, Happymind Inc., Gwacheon-shi, Gyeonggi-do, Republic of Korea; ^2^ Department of Psychiatry, Sanggye Paik Hospital, Inje University College of Medicine, Seoul, Republic of Korea; ^3^ Department of Psychiatry, Seoul National University College of Medicine & Seoul Metropolitan Government - Seoul National University (SMG-SNU) Boramae Medical Center, Seoul, Republic of Korea; ^4^ Department of Psychiatry, Seoul Brain Research Institute, Seoul, Republic of Korea; ^5^ Department of Teacher Education, College of Education, Konkuk University, Seoul, Republic of Korea

**Keywords:** ADHD, diagnostic validity, machine learning, attention test, comorbid psychiatric conditions

## Abstract

**Background:**

The diagnosis of attention deficit hyperactivity disorder (ADHD) relies on comprehensive approaches, including clinical interviews, scales, and neuropsychological assessments. However, the process is often limited by issues of reliability and availability.

**Objective:**

This study aims to develop a robust machine learning (ML) model using large-scale data from the Comprehensive Attention Test (CAT) to predict ADHD diagnoses.

**Methods:**

A total of 11,429 participants were recruited across South Korea and underwent the CAT. Of these, 7,737 were diagnosed with ADHD, including 6,772 with comorbid psychiatric conditions. Additionally, 850 individuals were included as a normal comparison group. Eight ML models were trained on a balanced dataset and validated using 5-fold cross-validation.

**Results:**

The CAT, when combined with the ML model, achieved an accuracy exceeding 0.98 in distinguishing pure ADHD cases from normal comparison groups. Classification accuracy was particularly high when distinguishing ADHD with comorbid externalizing disorders from normal control groups, especially in cases with more severe ADHD symptoms.

**Conclusion:**

The findings of this study suggest that the CAT, integrated with machine learning models, could serve as a promising tool for diagnosing ADHD.

## Introduction

Attention deficit hyperactivity disorder (ADHD) is a neurodevelopmental disorder characterized by persistent patterns of inattention, hyperactivity, and impulsivity, which lead to significant functional impairment across various environments, including home, school, and the workplace (1). ADHD affects approximately 5–7% of children worldwide, with 60–70% of these individuals continuing to exhibit symptoms into adulthood (2, 3). In South Korea, the prevalence is estimated to range from 2% to 5% among school-aged children, reflecting a similar trend observed globally (4).

Traditionally, the diagnosis of ADHD has relied on clinical interviews conducted by physicians, with accuracy and reliability often influenced by the clinician’s individual expertise and experience (5). To address these limitations, recent studies have focused on identifying ADHD biomarkers using neuroimaging, genetic data, and cognitive performance metrics, exploring diagnostic accuracy through machine learning (ML) techniques. Luo et al. (6) demonstrated that ensemble learning techniques applied to neuroimaging data improved ADHD classification, achieving a diagnostic accuracy of 0.89. Similarly, data from assessment questionnaires, when combined with a random forest-based ML model derived from the National Survey of Children’s Health, enabled early detection of ADHD with a reported diagnostic accuracy of 0.86 (7). Furthermore, neurocognitive assessments employing the continuous performance test (CPT) and ML models have predicted ADHD with a sensitivity of 0.89 and a specificity of 0.84 (8).

However, these studies faced significant limitations, such as potential overfitting due to small sample sizes, inconsistent and unbalanced datasets, and a lack of consideration for comorbid conditions, which could affect overall model performance. While there remains a demand for more accurate and practical models for ADHD diagnosis, the application of ML-based diagnostic tools faces substantial challenges, including high costs, procedural complexity, and limited sample sizes that hinder widespread adoption. Additionally, integrating ML models into clinical practice requires careful evaluation of ethical considerations and clinical effectiveness.

The Comprehensive Attention Test (CAT) offers a comprehensive and reliable assessment by evaluating various domains of attention. Previous studies have demonstrated the CAT’s reliability as a diagnostic tool for ADHD, highlighting its ability to detect detailed attention patterns that are critical characteristics of the disorder (9, 10). Recent research has further validated the CAT’s utility as a diagnostic tool, achieving diagnostic accuracy rates of 0.86 in children, 0.85 in adolescents, and 0.77 in adults with ADHD (11).

This study aims to develop an ML model for diagnosing ADHD using the CAT, leveraging a large sample size to address existing limitations. Additionally, we considered factors such as ADHD symptom severity and comorbidities that influence the clinical manifestation and diagnosis of ADHD. By utilizing a larger dataset and accounting for these factors, we anticipated improvements in the accuracy and reliability of ADHD diagnosis.

## Method

The research was conducted in accordance with the ethical standards outlined in the Helsinki Declaration. The ethics review board of Sanggye Paik Hospital approved the study protocol (IRB no. SGPAIK 2024-04-015). Informed consent (and assent, where applicable) was obtained from all participants, and for participants under the age of legal consent, parental consent was also secured.

### Participants

A total of 11,429 patients were recruited from 15 clinics across South Korea. The cohort included 10,144 individuals who presented with primary subjective complaints or objective symptoms of inattention and/or hyperactivity/impulsivity and underwent the CAT assessment between June 2017 and June 2018. Additionally, data collected for the standardization of the CAT from February to April 2008 were included in the normal comparison group.

Individuals with intellectual disabilities, as confirmed by the Korean version of the Wechsler Intelligence Scale for Children-IV (K-WISC-IV), were excluded from the study (n = 641). To ensure accuracy of administration, K-WISC-IV was conducted by qualified and licensed clinical psychologists who have taken clinical practices and supervisions in authorized training mental hospitals for at least 3 years.

Among the 7,737 participants diagnosed with ADHD, 6,772 had comorbid conditions, while 965 were diagnosed with pure ADHD. Among those with ADHD and other psychiatric disorders, 2,408 had externalizing disorders such as oppositional defiant disorder (ODD) or conduct disorder (CD), 2,897 had internalizing disorders such as depressive or anxiety disorders, and 1,485 had both.

**Figure 1:**
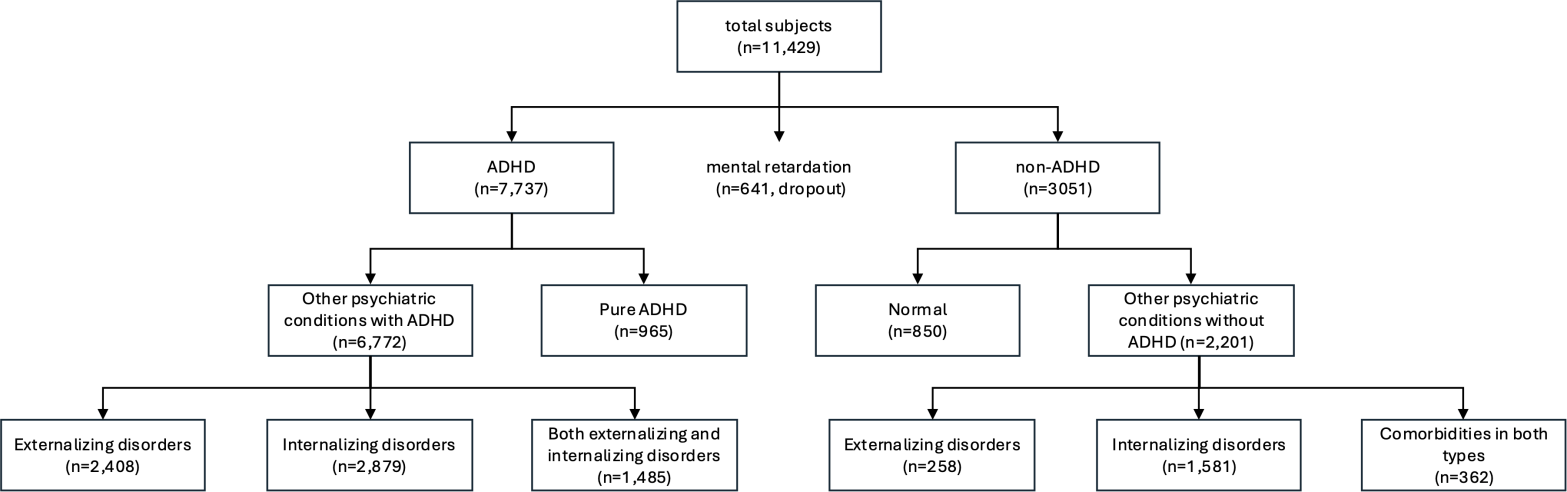
Diagram depicting the characteristics of the dataset. ADHD, attention deficit hyperactivity disorder.

Of the 3,051 participants without ADHD, 2,201 had concurrent psychiatric conditions, while 850 were free of psychiatric disorders. Among those with other psychiatric conditions, 258 had externalizing disorders, 1,581 had internalizing disorders, and 362 had both (**Figure 1**). All psychiatric diagnoses were made according to the Diagnostic and Statistical Manual of Mental Disorders (DSM-5) criteria, and the severity of illness was determined by certified child and adolescent psychiatrists who had been trained in training hospitals after acquiring a Korean Board of Psychiatry. The intraclass correlation for psychiatric diagnoses among the 15 psychiatrists was 0.73 (p <.001).

### Assessment

The CAT used in this study for ADHD diagnosis comprises six distinct subtests evaluating various aspects of attention and related cognitive functions. The total administration time was approximately 50-60 minutes on average. The test was administered individually on a computer by a trained research assistant who provided standardized instructions and demonstrations. Specific details of each subtest are summarized in **Supplementary Table S1**.

- Visual Selective Attention (VSA) and Auditory Selective Attention (ASA): Measure the ability to focus on and select visual or auditory stimuli.
- Sustained Attention (SA): Assesses the ability to maintain or suppress responses to repeated stimuli.
- Interference Selective Attention (ISA) (Flanker Test): Evaluates the ability to focus on relevant information while ignoring distractions. Forward stimuli present target and distractor cues in the same direction, whereas backward stimuli involve opposing directional cues.
- Divided Attention (DA): Measures the ability to process visual and auditory stimuli simultaneously.
- Spatial Working Memory (WM): Assesses the ability to temporarily retain and recall visuospatial sequences in forward and reverse order.

The measured variables used in model development included.

- Omission Errors (OE): Frequency of failing to respond to target stimuli.
- Commission Errors (CE): Frequency of responding to non-target stimuli.
- Mean Reaction Time: Average of correct response times to target stimuli.
- Standard Deviation of Reaction Time: Variability of response times to target stimuli.
- d’ (Sensitivity Index): Measures the ability to distinguish between target and non-target stimuli. Higher scores indicate better discrimination.
- b (Beta): Relative proportion of CE to OE, indicating response style.

For each measured variable, the attention quotient (AQ) was calculated by standardizing the variable to a mean of 100 and a standard deviation of 15, based on previous standardization studies (12).

### Data preprocessing

CAT components differ by age group, resulting in potential missing scores for some participants. Missing data were imputed using the median value of the overall distribution. The Synthetic Minority Over-Sampling Technique (SMOTE) was applied to balance the class labels in the test dataset (13). SMOTE effectively mitigates bias in imbalanced datasets by generating synthetic samples, thereby enhancing the classifier’s ability to identify minority class instances (14). Scale normalization was also performed to standardize the data for machine learning analysis.

### Machine learning model

We trained and evaluated eight supervised algorithms: four non-ensemble learners (Naïve Bayes, k-nearest neighbours, Decision Tree, and Support Vector Machine) and four ensemble learners (Random Forest, Gradient Boosting, LightGBM, and CatBoost). Non-ensemble methods generate predictions from a single decision function, making them computationally lightweight and comparatively easy to interpret. Ensemble methods, in contrast, combine the outputs of many weak learners—either simultaneously (bagging) or sequentially (boosting)—to reduce variance and bias, and they often achieve higher predictive accuracy.

Employing both families served two objectives. First, it enabled a direct performance comparison between simple, interpretable baselines and state-of-the-art ensembles—relevant because ensembles have repeatedly outperformed single models in disease classification studies. This comparative approach aligns with structured frameworks for evaluating multiple machine-learning models across varied datasets (15, 16). Second, it allowed us to quantify the trade-off between the incremental accuracy gained and the interpretability lost when clinicians adopt complex decision-support tools. To cover the full spectrum of data dimensionality, feature interactions, deployment constraints, and transparency requirements, we selected algorithms that span diverse inductive biases, in line with prior work that structured model comparisons for applied decision support tasks (17). A concise description of all eight algorithms is provided in **Supplementary Table S2**. Feature importance was determined using a gradient boosting regression model, which provided insights into the relative significance of each feature in predicting outcomes. A training dataset of 850 normal control individuals and 965 individuals with pure ADHD was used to construct the classification model.

The training and test datasets were validated using 5-fold cross-validation. To analyze ADHD classification results based on comorbid conditions, the training dataset was further segmented by comorbid type (externalizing or internalizing) and ADHD severity (mild or moderate to severe). Metrics such as F1-score, Macro Precision, Macro Recall, and Macro F1-score were reported to evaluate the model’s ability to distinguish between different disease classes, ensuring a balanced assessment across all groups.

All ML model development and statistical analyses were performed using Python 3 (version 3.9.19) with the scikit-learn, CatBoost, and LightGBM libraries.

## Results

### Demographic data

Among the normal control group, 435 (51.18%) were male and 415 (48.82%) were female, with an average age of 13.97 years. In the pure ADHD group, 822 (74.86%) were male and 276 (25.14%) were female, with an average age of 11.37 years. Among subjects diagnosed with other psychiatric conditions along with ADHD, 5,373 (74.26%) were male and 1,862 (25.74%) were female, with an average age of 10.93 years. In the group with other psychiatric conditions but without ADHD, 1,601 (72.74%) were male and 600 (27.26%) were female, with an average age of 11.40 years.

Due to significant age differences between the groups (F = 82.034; p <.001), standardized scores derived from prior research encompassing children and adults were used for subsequent analyses.

### Diagnostic validity of the CAT using the ML model

Using the CAT, we developed a model capable of distinguishing between pure ADHD and normal comparison groups with an accuracy exceeding 0.98. This high performance was most evident in ensemble models, particularly the gradient boosting model (**Table 1**).

**Table 1:** Model evaluation results for distinguishing between pure attention deficit hyperactivity disorder (N = 965) and normal comparison group (N = 850).

Machine learning model	Accuracy	Sensitivity	Specificity	F1-score	Macro precision	Macro recall	Macro f1-score
non–ensemble learning							
naive bayes	0.76	0.71	0.84	0.78	0.76	0.77	0.76
k-nearest neighbor	0.88	0.84	0.94	0.89	0.88	0.89	0.88
decision tree	0.91	1.00	0.77	0.93	0.93	0.89	0.90
vvsupport vector machine	0.96	0.95	0.98	0.96	0.96	0.96	0.96
ensemble learning							
catboost	0.87	1.00	0.71	0.89	0.90	0.85	0.86
random forest	0.89	1.00	0.72	0.91	0.92	0.86	0.88
gradient boosting	0.98	0.99	0.96	0.98	0.98	0.98	0.98
lightGBM	0.94	1.00	0.86	0.95	0.95	0.93	0.93

In our investigation of metrics for model generation, we found that the Flanker subtest emerged as the primary indicator for distinguishing pure ADHD from normal control groups (**Supplementary Table S3**). A classification model utilizing only this metric achieved an impressive accuracy of 0.92. Conversely, the working memory metric contributed the least to model generation (**Table 2**).

**Table 2:** Model evaluation results comparing pure attention deficit hyperactivity disorder (N = 965) and normal comparison groups (N = 850) across subtests of the comprehensive attention test.

Subtests	Accuracy	Sensitivity	Specificity	F1-score	Macro precision	Macro recall	Macro f1-score
visual selective attention	0.81	0.87	0.73	0.84	0.81	0.80	0.80
auditory selective attention	0.78	0.83	0.70	0.82	0.77	0.77	0.77
sustained attention to response	0.79	0.82	0.76	0.81	0.79	0.79	0.79
flanker	0.92	0.87	0.98	0.92	0.92	0.92	0.92
divided attention	0.71	0.82	0.58	0.76	0.72	0.70	0.70
spatial working memory	0.62	0.77	0.42	0.70	0.60	0.59	0.59

We further explored whether the CAT could differentiate ADHD based on comorbidity and symptom severity, which are closely related to the clinical and neuropsychological features of ADHD. The classification performance of the CAT improved in cases with greater ADHD severity and comorbid externalizing disorders. For example, the diagnostic accuracy for mild ADHD and moderate-to-severe ADHD with externalizing disorders was 0.89 and 0.94, respectively. However, the model’s accuracy decreased for ADHD cases with internalizing disorders. Additionally, when the model was applied to distinguish between ADHD with comorbidities and pure ADHD, the classification accuracy was lower (**Table 3**).

**Table 3:** Classification model performance comparisons based on symptom severity and comorbidity type in attention deficit hyperactivity disorder, + C: with comorbidities, - C: without comorbidities, ± C: with and without comorbidities, - ADHD: without ADHD.

**Comorbid type**	**ADHD severity**	**Accuracy**	**Precision**	**Recall**	**Macro f1**
Externalizing					
	Mild (28.795)				
	ADHD ± C vs. non-ADHD ± C	0.89	0.87	0.87	0.87
	ADHD + C vs. ADHD - C	0.56	0.52	0.52	0.51
	Moderate to Severe (6.763)				
	ADHD ± C vs. non-ADHD ± C	0.94	0.94	0.95	0.94
	ADHD + C vs. ADHD - C	0.63	0.47	0.45	0.45
Internalizing					
	Mild (35.189)				
	ADHD ± C vs. non-ADHD ± C	0.74	0.77	0.69	0.69
	ADHD + C vs. ADHD – C	0.56	0.49	0.49	0.49
	Moderate to Severe (7.324)				
	ADHD ± C vs. non-ADHD ± C	0.76	0.72	0.74	0.73
	ADHD + C vs. ADHD - C	0.71	0.46	0.47	0.46

**Figure 2:**
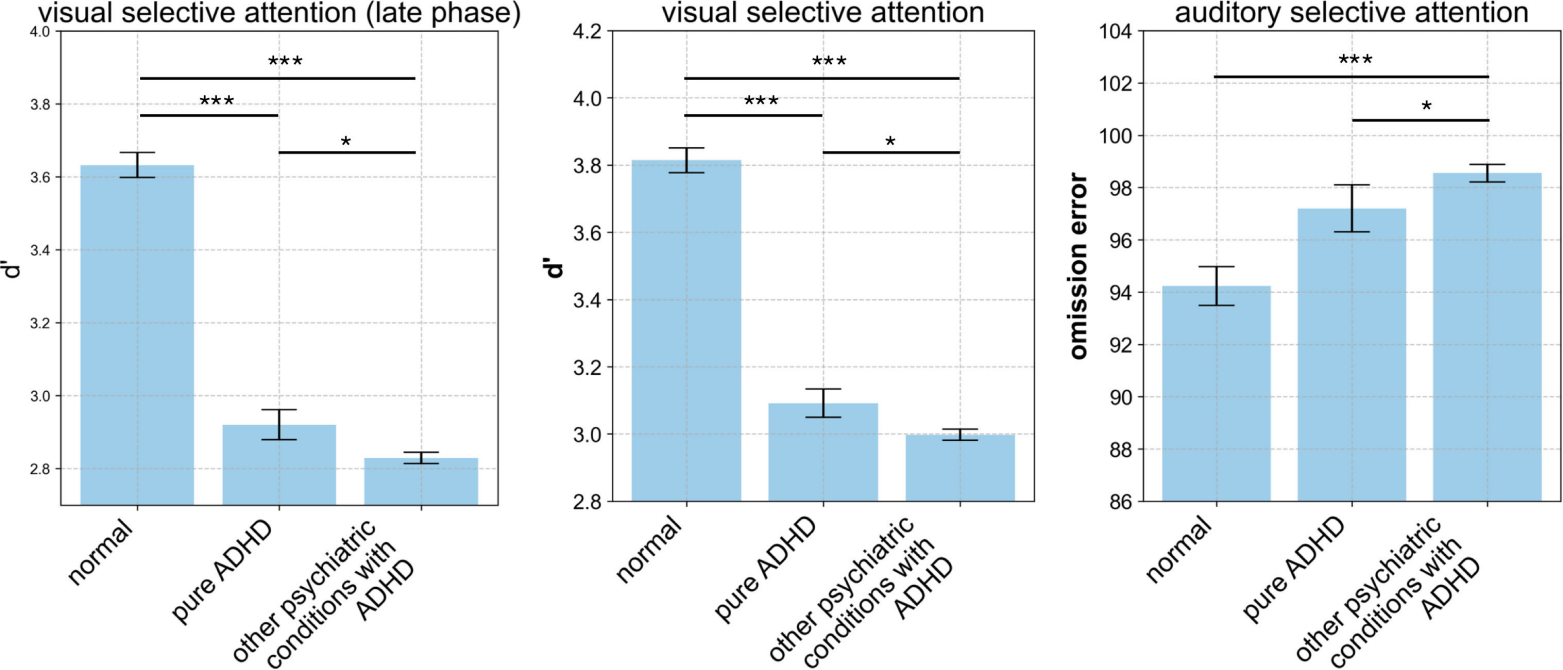
Comparison of CAT Performance Among Normal Control, Pure ADHD, and Comorbid Groups. The comparison of CAT performance among groups was conducted using one-way ANOVA, followed by independent t-tests for *post-hoc* analysis with Bonferroni correction. Significance levels are indicated as follows: *p < 0.05, ***p < 0.001. CAT, comprehensive attention test; ADHD, attention deficit hyperactivity disorder; ANOVA, analysis of variance.

Statistical analyses of CAT scores were conducted according to the presence of comorbid conditions in the ADHD group (**Figure 2**). Individuals with ADHD showed reduced d’ scores compared to the normal group in the VSA subtest, with a more pronounced decline under comorbid conditions, particularly during the latter 50% of the test. In the ASA subtest, individuals with ADHD committed a higher number of OE than the normal group, with an increased number of errors observed under comorbid conditions.

## Discussion

In this study, we observed high accuracy in the CAT when combined with our machine learning (ML) models, particularly the gradient boosting model, which achieved an accuracy exceeding 0.98. These findings suggest that the CAT, with its comprehensive assessment of various cognitive domains, can serve as a reliable tool for diagnosing ADHD, potentially surpassing previous assessment methods. Unlike earlier studies that faced limitations such as small sample sizes, inconsistent and unbalanced datasets, and limited consideration of comorbid conditions, our approach offers greater applicability and usability in clinical settings (6, 7). Notably, the Flanker test emerged as a critical attention test for discriminating ADHD, achieving an accuracy of 0.92.

One of the primary strengths of this study is its large sample size, which enhances the generalizability of the findings. The inclusion of a nationwide cohort, spanning various age groups and comorbid conditions, ensures that the model captures diverse demographic and clinical manifestations of ADHD. Additionally, by testing eight different ML models, including both non-ensemble and ensemble approaches, we identified effective models for ADHD diagnosis.

Moreover, the study demonstrated improved classification performance in ADHD cases with higher symptom severity and comorbid externalizing disorders. These results align with previous findings, which indicate that the diagnosis of ADHD is influenced by symptom severity and comorbidity. Our model was particularly effective in cases with severe ADHD symptoms and comorbid conditions such as ODD or CD (18–20).

Previous research supports the notion that higher ADHD severity is strongly associated with more externalizing symptoms, such as hyperactivity and impulsivity. In contrast, individuals with milder ADHD symptoms or predominant internalizing symptoms, such as anxiety or depression, are less likely to exhibit overt behavioral patterns, complicating the diagnostic process (18, 19). Additionally, the complex attentional deficits linked to comorbid conditions, such as depression and anxiety, make diagnostic classification more challenging (20). The lower classification accuracy in distinguishing between ADHD with and without comorbidities indicates the need for further refinement of the model. To improve accuracy, future analyses should focus on specific psychiatric disorders rather than broadly categorized conditions, incorporating more diverse and numerous cases.

The results also revealed performance differences in individuals with ADHD, particularly in the presence of comorbid conditions. Individuals with ADHD exhibited reduced target/non-target discrimination abilities compared to the normal group in the VSA subtest, with more pronounced impairment under comorbid conditions during the latter 50% of the test. This suggests that comorbid conditions exacerbate difficulties in maintaining VSA over time.

Furthermore, in the ASA subtest, individuals with ADHD committed a higher number of OE than the normal group, with even more errors observed in those with comorbid conditions. This finding underscores the additional challenges faced by individuals with both ADHD and comorbid conditions in processing auditory attention tasks.

The VSA and ASA tests, which measure vigilance under conditions of low target frequency, are particularly challenging for distinguishing individuals with ADHD from those without ADHD. Decreased discrimination ability during the latter stage of the VSA subtest or an increased number of OEs in the ASA subtest is more evident in patients with ADHD and concurrent psychiatric disorders, who exhibit more severe attention impairments compared to those with pure ADHD (21–23).

The inclusion of only South Korean participants may limit the generalizability of the findings, emphasizing the need for future research to incorporate multi-ethnic data for a more universally applicable model. Furthermore, considering the specific types and severity of comorbid conditions will be crucial for enhancing the robustness of ADHD diagnoses. Increasing the sample size will also improve the precision and reliability of the classification model.

## Conclusion

The findings of this study highlight the feasibility of using the CAT in combination with ML models as a reliable tool for diagnosing ADHD. The effectiveness is particularly notable in cases of higher ADHD symptom severity and comorbid externalizing disorders. However, it is crucial to emphasize that the results from this tool must be interpreted within the context of a full clinical evaluation. The value of this instrument lies not in functioning as a standalone diagnostic test, but in providing adjunctive objective data to the clinician. Future research should explore how CAT results can be optimally integrated into the multifaceted clinical decision-making process.

## Data Availability

The raw data supporting the conclusions of this article will be made available by the authors, without undue reservation.
