# Supplementary material for: Prediction of attention deficit hyperactivity disorder using the comprehensive attention test: a large-scale machine learning approach
Source: Front Psychiatry. 2025 May 27;16:1574615. doi: 10.3389/fpsyt.2025.1574615 (PMC12149198; doi:10.3389/fpsyt.2025.1574615)
Supplement: Supplementary file 1 [file Table1.docx]

| **Subtest Name** | **Task Description and Administration** |
| --- | --- |
| Selective Attention (Visual and Auditory) | Measures the ability to focus on target stimuli and ignore non-targets in a single modality. In the visual selective attention task, for example, the participant watches a sequence of stimuli on a screen and must quickly press a button whenever a specific target shape (circle) appears among other shapes. In the auditory version, the participant listens via headphones and responds when a target sound (bell) is heard among distractor sounds. These subtests evaluate how well the participant can select relevant information while filtering out distractions. |
| Sustained Attention | Assesses the ability to maintain attention and inhibitory control over a prolonged period. In this go/no-go style task, the participant is instructed to respond to frequent “go” stimuli but withhold responses when an infrequent “no-go” stimulus appears (for example, press the button for any letter except the letter “X”). This evaluates the capacity to sustain focus and suppress impulsive responses to the repeating stimuli |
| Interference-Selective Attention (Flanker Task) | Measures the ability to selectively attend to a target stimulus while ignoring interference from distractors. In this subtest (modeled on the Flanker test), the participant sees a row of stimuli (direction of the open side of the middlebox) and must respond to the central target stimulus’s direction while ignoring the flanking stimuli which may be congruent or incongruent distractors. This tests how well one can resist distraction and process relevant information in the presence of competing inputs. |
| Divided Attention | Evaluates the ability to process information from two modalities simultaneously. Here, the participant must attend to both visual and auditory targets presented together. For example, the participant might be required to monitor a visual sequence and an auditory sequence at the same time, responding whenever either a specific image appears on screen or a specific sound plays. This dual-task condition measures multitasking ability and the allocation of attention across modalities. |
| Spatial Working Memory | Assesses the ability to temporarily hold and manipulate information (visuospatial memory). In this subtest, a series of spatial locations or patterns is presented (e.g., sequences of blocks lighting up on the screen), and the participant must recall the sequence either in the same order or in reverse order after a brief delay. This tests short-term memory capacity and the executive aspect of updating/manipulating stored information. |

**Table S1.** Detailed information on the computerized comprehensive attention test.

| **Model Name** | **Type** | **Key Characteristics / Principle** |
| --- | --- | --- |
| Naive Bayes | Non-Ensemble | Probabilistic classifier that applies Bayes’ theorem with a *naïve* assumption of conditional independence among features. Extremely fast and performs well on high-dimensional, sparse data. |
| k-Nearest Neighbors (k-NN) | Non-Ensemble | Instance-based, non-parametric method. Assigns class labels by majority vote among the *k* closest samples in feature space. Very simple but sensitive to feature scaling and the curse of dimensionality. |
| Decision Tree | Non-Ensemble | Recursively partitions the feature space into axis-aligned splits that minimize an impurity criterion. Highly interpretable yet prone to over-fitting unless depth or leaf size is constrained. |
| Support Vector Machine (SVM) | Non-Ensemble | Margin-based classifier that finds the hyperplane maximising class separation; kernel trick allows non-linear boundaries. Works well in high-dimensional spaces but requires careful hyper-parameter tuning. |
| CatBoost | Ensemble (Boosting) | Gradient-boosting framework with *ordered boosting* and *target-based statistics* that minimise prediction shift for categorical variables. Requires minimal preprocessing, reduces over-fitting, and delivers strong out-of-the-box accuracy on heterogeneous tabular data. |
| Random Forest | Ensemble (Bagging) | Constructs many decorrelated decision trees on bootstrap samples and random feature subsets; aggregates predictions by majority vote or averaging. Reduces variance, controls over-fitting, and provides feature-importance scores. |
| Gradient Boosting Machine (GBM) | Ensemble (Boosting) | Builds shallow trees sequentially, each correcting the residuals of the previous ensemble via gradient-descent on a differentiable loss. Often yields high accuracy but can over-fit without learning-rate and early-stopping controls. |
| Light Gradient Boosting Machine (LightGBM) | Ensemble (Boosting) | High-performance GBM implementation that uses histogram-based, leaf-wise tree growth and gradient-based one-side sampling. Handles very large, sparse, or high-dimensional data efficiently and supports native categorical splits. |

**Table S2.** Machine Learning Models Employed.

| **Variable** | **Feature importance** |
| --- | --- |
| flanker (forward) *beta* | 59.84 |
| flanker (forward) *d* | 15.02 |
| flanker omission error *AQ* | 6.57 |
| flanker *d* | 6.10 |
| auditory selective attention *beta* | 1.94 |
| sustained attention to response omission error *AQ* | 1.33 |
| divided attention *beta* | 1.13 |
| visual selective attention (early phase) *d* | 0.94 |
| flanker true RT mean *AQ* | 0.87 |
| auditory selective attention omission error *AQ* | 0.55 |
| sustained attention to response commission error *AQ* | 0.38 |
| flanker (backward) *beta* | 0.36 |
| visual selective attention (late phase) *d* | 0.36 |
| visual selective attention RT std *AQ* | 0.35 |
| flanker commission error *AQ* | 0.29 |
| auditory selective attention commission error *AQ* | 0.29 |
| auditory selective attention RT std *AQ* | 0.29 |
| sustained attention to RT std *AQ* | 0.29 |
| sustained attention to response *beta* | 0.25 |
| flanker *beta* | 0.24 |

**Table S3.** Multivariable Feature Importance in the Gradient Boosting Decision Tree Model Comparing Pure Attention Deficit Hyperactivity Disorder (N = 965) and Normal Comparison Groups (N = 850). RT: response time, AQ: attention quotient.
